# Supplementary material for: Cyanotoxins Increase Cytotoxicity and Promote Nonalcoholic Fatty Liver Disease Progression by Enhancing Cell Steatosis
Source: Toxins (Basel). 2023 Jun 25;15(7):411. doi: 10.3390/toxins15070411 (PMC10467139; doi:10.3390/toxins15070411)
Supplement: Supplementary file 1 [file toxins-15-00411-s001.zip › toxins-2372558-supplementary.pdf]

Figure S1

Microcystin-LR

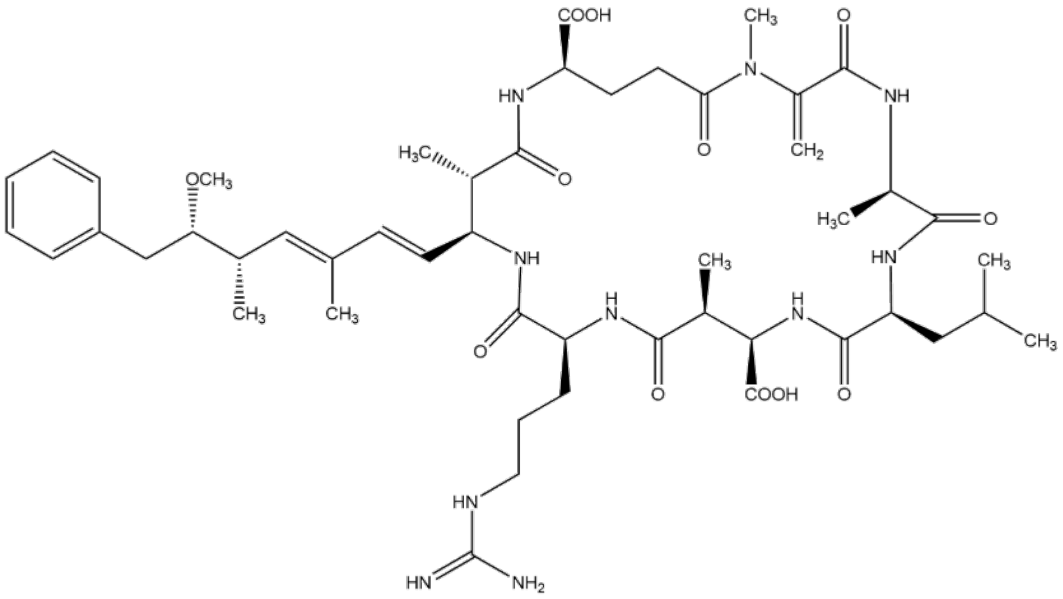

Microcystin-RR

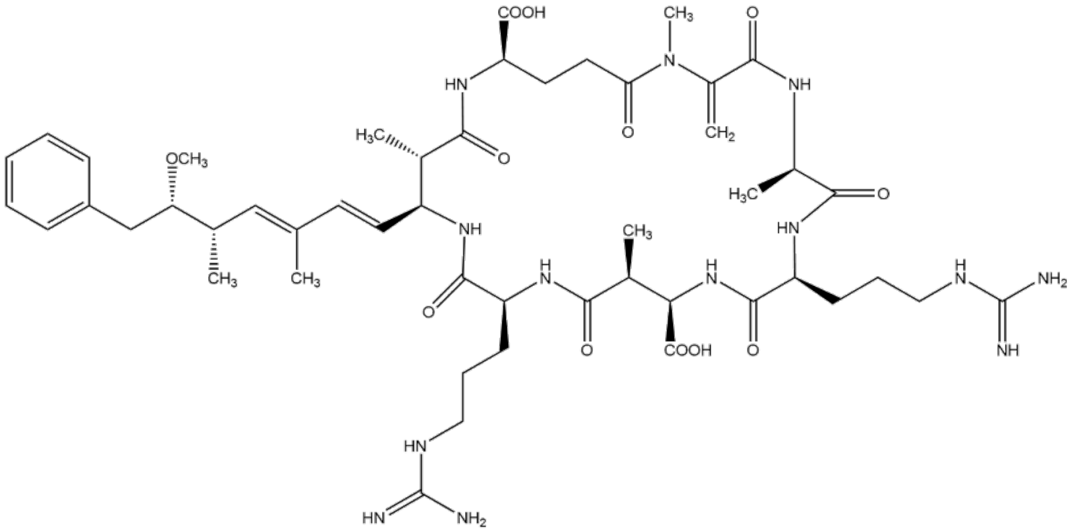

Nodularin

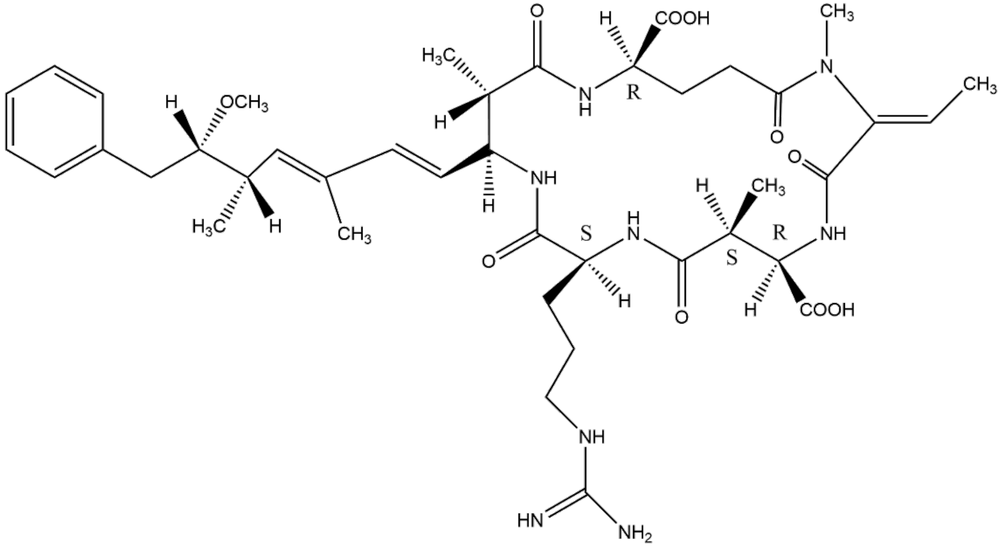

Cylindrospermopsin

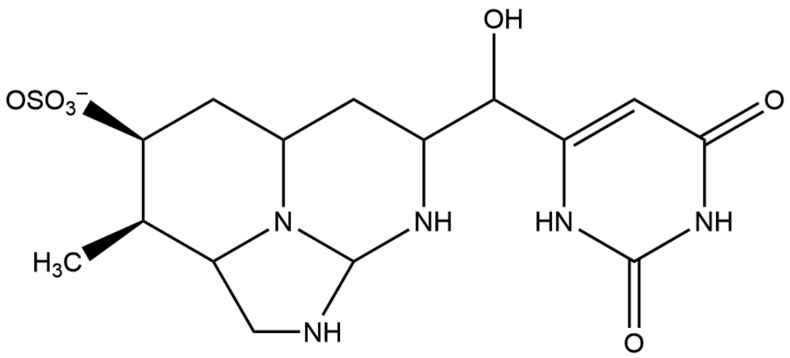

Figure S1. Chemical structures of cyanotoxins. Chemical structures of cyanotoxins used in the current study such as microcystin -LR (MC-LR), microcystin-RR (MC-RR), nodularin (NOD), and cylindrospermopsin (CYN) were presented. Chemical structures were drawn by ChemDraw Professional (Perkin Elmar).

Figure S2

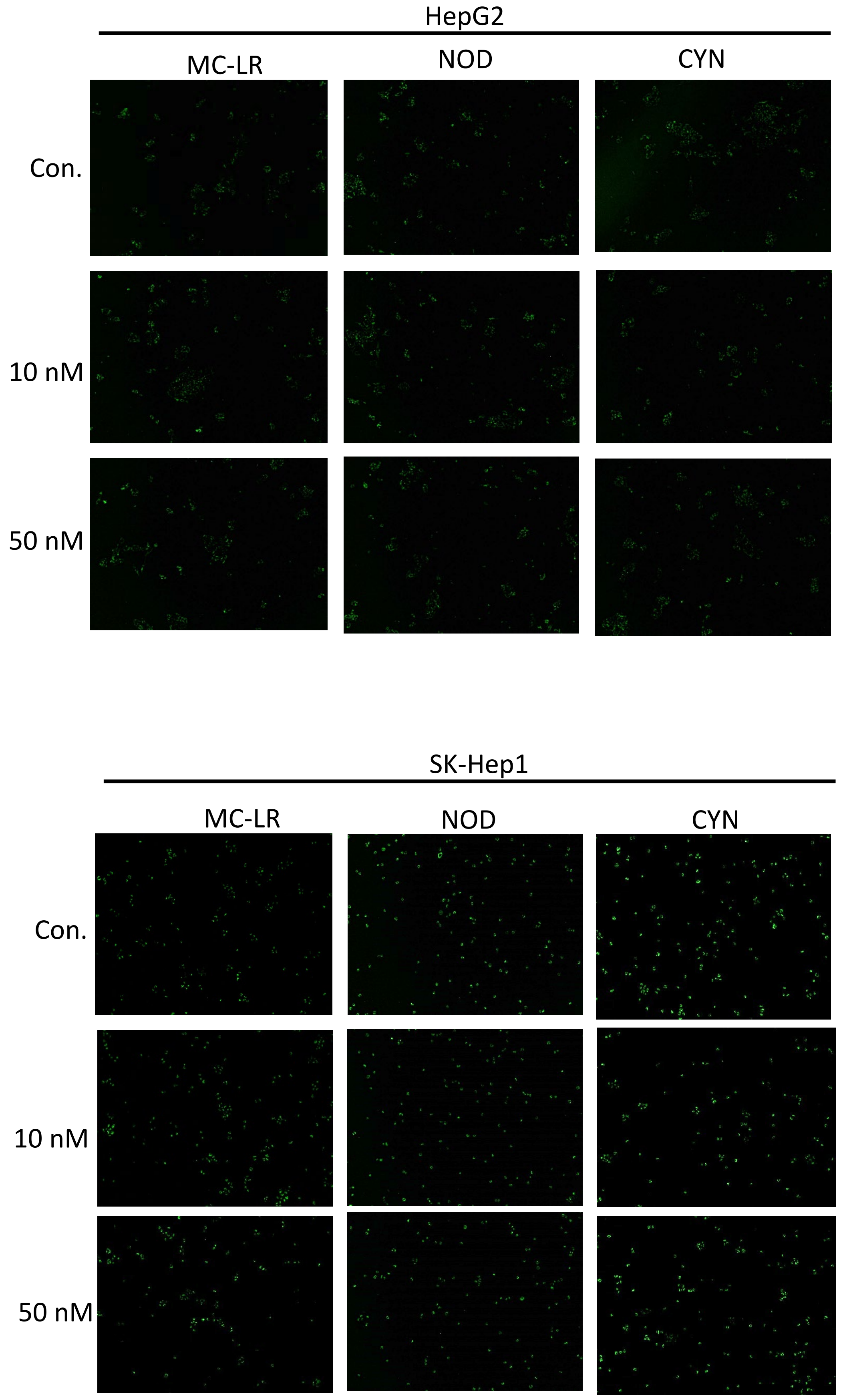

Figure S2. Effect of cyanotoxins on reactive oxygen species (ROS) production in HCC cells. HCC HepG2 and SK-Hep1cells were plated in 96 well plates for 18 h in triplicates and cells were treated with 10 and 50 nM concentrations of indicated cyanotoxins for 72 h. Cells were treated with CellRox green reagent, washed with PBS, and observed under Keyence BZX-810 fluorescence microscope (10× objective) and images were captured.

Supplementary Table S1

Effect of MC-LR exposure on top gene expression in human hepatocytes

Gene ID	Gene Name	Regulation	Log2 Fold-change	p Value
ENSG00000197632	SERPINB2	Down	-10.3482	3.60E-17
ENSG00000276043	UHRF1	Down	-8.69805	4.19E-11
ENSG00000178726	THBD	Down	-8.69237	3.76E-11
ENSG00000090382	LYZ	Down	-8.46236	2.04E-10
ENSG00000153162	BMP6	Down	-8.26095	1.21E-09
ENSG00000127080	IPPK	Down	-8.12224	4.32E-09
ENSG00000102271	KLHL4	Down	-8.11257	6.26E-09
ENSG00000204644	ZFP57	Down	-7.8196	3.82E-08
ENSG00000073756	PTGS2	Down	-7.5306	2.63E-07
ENSG00000214866	DCDC2C	Down	-7.52715	2.46E-07
ENSG00000101200	AVP	Up	8.974497	0.021662
ENSG00000269466	H3.Y	Up	8.296403	1.81E-08
ENSG00000261481	AC022167.4	Up	7.95965	1.71E-07
ENSG00000279428	AC087164.2	Up	7.958691	4.82E-09
ENSG00000272989	LINC02012	Up	7.874452	1.00E-08
ENSG00000259575	AC012404.2	Up	7.607555	3.98E-06
ENSG00000280306	AC137056.2	Up	7.479271	4.63E-07
ENSG00000279141	LINC01451	Up	7.448303	7.86E-06
ENSG00000204481	PRAMEF14	Up	7.405007	7.50E-06
ENSG00000284523	AC004834.1	Up	7.259207	1.92E-05

Effect of CYN exposure on top gene expression in human hepatocytes

Gene ID	Gene Name	Regulation	Log2 Fold-change	p Value
ENSG00000267909	CCDC177	Down	-9.15544	3.47E-13
ENSG00000127080	IPPK	Down	-8.85762	3.88E-12
ENSG00000119508	NR4A3	Down	-8.58032	3.11E-11
ENSG00000227473	TSSK5P	Down	-8.48377	1.25E-10
ENSG00000172967	XKR3	Down	-7.89389	7.15E-09
ENSG00000244932	AL449212.1	Down	-7.50561	1.42E-07
ENSG00000142178	SIK1	Down	-7.47415	5.14E-11
ENSG00000157654	PALM2-AKAP2	Down	-7.4169	2.63E-07
ENSG00000264268	MIR4767	Down	-7.4034	2.11E-07
ENSG00000204920	ZNF155	Down	-7.0787	1.76E-06
ENSG00000256955	AC131009.2	Up	7.511456	4.10E-06
ENSG00000255435	AP001267.3	Up	7.33209	1.15E-05
ENSG00000234504	AL353801.2	Up	7.303084	1.45E-05

ENSG00000103375	AQP8	Up	7.072418	4.94E-05
ENSG00000236512	AL390026.1	Up	6.924002	0.000193
ENSG00000161055	SCGB3A1	Up	6.922405	6.83E-06
ENSG00000243609	RPS2P44	Up	6.900767	0.000739
ENSG00000101200	AVP	Up	6.876104	0.023686
ENSG00000249551	LINC02216	Up	6.850483	0.003579
ENSG00000149435	GGTLC1	Up	6.819321	5.01E-08

Effect of MC-LR exposure on top gene expression in HepaRG cells

Gene ID	Gene Name	Regulation	Log2 Fold-change	p Value
ENSG00000257941	AC011611.5	Down	-5.76423	0.001855
ENSG00000272864	AC135803.1	Down	-5.58907	0.002511
ENSG00000183628	DGCR6	Down	-5.45996	0.001356
ENSG00000214435	AS3MT	Down	-5.44091	0.008949
ENSG00000228092	COX6CP15	Down	-5.3825	0.009732
ENSG00000254231	AC103760.1	Down	-5.366	0.010723
ENSG00000221540	MIR1180	Down	-5.3457	0.01145
ENSG00000243819	RN7SL832P	Down	-5.33707	0.005633
ENSG00000132965	ALOX5AP	Down	-5.29178	0.00636
ENSG00000273341	AC004921.1	Down	-5.27057	0.013477
ENSG00000277170	AC012676.3	Up	5.279552	0.002157
ENSG00000235910	APOA1-AS	Up	4.889909	0.007552
ENSG00000268066	FMR1-AS1	Up	4.769747	0.014786
ENSG00000249098	AC055733.1	Up	4.746104	0.026444
ENSG00000112212	TSPO2	Up	4.729245	0.015812
ENSG00000124507	PACSIN1	Up	4.641328	0.030097
ENSG00000104804	TULP2	Up	4.636511	0.033402
ENSG00000267560	AC027514.2	Up	4.631242	0.016421
ENSG00000239627	RPL12P20	Up	4.614073	0.034225
ENSG00000262213	AC144836.1	Up	4.575241	0.038558

Effect of CYN exposure on top gene expression in HepaRG cells

Gene ID	Gene Name	Regulation	Log2 Fold-change	p Value
ENSG00000255965	AC073916.1	Down	-5.60243	0.001474
ENSG00000021488	SLC7A9	Down	-5.57866	0.001308
ENSG00000213658	LAT	Down	-5.53477	0.0016
ENSG00000243498	UBA52P5	Down	-5.45298	0.002962
ENSG00000257941	AC011611.5	Down	-5.34091	0.003463
ENSG00000275263	AC135048.3	Down	-5.2499	0.004249
ENSG00000225857	AL162431.1	Down	-5.20917	0.004708

ENSG00000251455	AC092611.1	Down	-5.20663	0.006018
ENSG00000242616	GNG10	Down	-5.14758	0.005808
ENSG00000271754	AL355802.2	Down	-5.13248	0.006678
ENSG00000267302	RNFT1-DT	Up	5.67702	0.001192
ENSG00000230042	AK3P3	Up	5.335992	0.000494
ENSG00000112212	TSPO2	Up	5.228155	0.005058
ENSG00000166148	AVPR1A	Up	5.109601	0.012102
ENSG00000254008	LINC00051	Up	5.086984	0.01069
ENSG00000224525	AL591686.1	Up	4.902105	0.002496
ENSG00000185681	MORN5	Up	4.898449	0.013276
ENSG00000176532	PRR15	Up	4.866438	0.045555
ENSG00000274308	AC244093.1	Up	4.85936	0.012708
ENSG00000276747	PADI6	Up	4.856956	0.017482

Effect of MC-LR exposure on top gene expression in HCC HepG2 cells

Gene ID	Gene Name	Regulation	Log2 Fold-change	p Value
ENSG00000227726	AP001271.1	Down	-5.77119	0.000247
ENSG00000224621	AL451042.1	Down	-5.07446	0.003605
ENSG00000279337	AC127024.7	Down	-5.04122	0.004025
ENSG00000179938	GOLGA8J	Down	-4.89031	0.009243
ENSG00000270429	KNOP1P2	Down	-4.86232	0.007469
ENSG00000205424	AL592528.1	Down	-4.7868	0.009163
ENSG00000266980	AC087289.1	Down	-4.75391	0.019707
ENSG00000122122	SASH3	Down	-4.75164	0.037803
ENSG00000271924	RNA5SP108	Down	-4.6944	0.026239
ENSG00000280291	AC016251.2	Down	-4.6918	0.013511
ENSG00000237001	WASF3-AS1	Up	5.113503	0.003383
ENSG00000206199	ANKUB1	Up	4.980121	0.005032
ENSG00000271819	RNU6-94P	Up	4.878079	0.006998
ENSG00000227527	AC096540.1	Up	4.866142	0.007704
ENSG00000236545	AP001619.2	Up	4.846916	0.007804
ENSG00000273973	AC025162.2	Up	4.749642	0.003784
ENSG00000225423	TNPO1P1	Up	4.734482	0.010625
ENSG00000144668	ITGA9	Up	4.567547	0.018566
ENSG00000131386	GALNT15	Up	4.545009	0.035267
ENSG00000250327	RPSAP70	Up	4.528042	0.018918

Effect of CYN exposure on top gene expression in HCC HepG2 cells

Gene ID	Gene Name	Regulation	Log2 Fold-change	p Value

ENSG00000250057	AC114781.2	Down	-5.2059	0.00216
ENSG00000279613	AC124283.5	Down	-4.92677	0.007393
ENSG00000236577	SNRPGP14	Down	-4.87974	0.013408
ENSG00000175877	TMEM270	Down	-4.81511	0.009601
ENSG00000261644	AC007728.2	Down	-4.73239	0.010795
ENSG00000094963	FMO2	Down	-4.71905	0.011325
ENSG00000187479	C11orf96	Down	-4.71027	0.015791
ENSG00000149646	CNBD2	Down	-4.67315	0.016098
ENSG00000241429	EEF1A1P25	Down	-4.62129	0.019465
ENSG00000207750	MIR553	Down	-4.61098	0.014386
ENSG00000273973	AC025162.2	Up	5.785751	0.000479
ENSG00000140832	MARVELD3	Up	4.977276	0.004972
ENSG00000200966	RN7SKP87	Up	4.773934	0.010473
ENSG00000221227	MIR1305	Up	4.687057	0.012914
ENSG00000250889	LINC01336	Up	4.630872	0.028731
ENSG00000227091	AL355990.1	Up	4.618517	0.018966
ENSG00000265018	AGAP12P	Up	4.612642	0.00374
ENSG00000169605	GKN1	Up	4.610885	0.026522
ENSG00000232290	AL133260.2	Up	4.6025	0.04812

Supplementary Table S2

Set of primers used in the current study.

Primer sequence
GAPDH FP: 5’-CCACCCAGAAGACTGTGGAT -3’<br>GAPDH RP: 5’-GTTGAAGTCAGAGGAGACCACC-3’
IRE1a FP: 5’-GTGGCCTTCATCATCACCTATC-3’<br>IRE1a RP: 5’-CCTCATCTCCATCGTCTTGTTC-3’
eIF2a FP: 5’-CCTTCTGCTGCCTCTAAGATAAA-3’<br>eIF2a RP: 5’-GGGAGCTGAGTGCTACAATAAA-3’
ATF4 FP: 5’-CCATGATCCCTCAGTGCATAA-3’<br>ATF4 RP: 5’-TGCGGACCTCTTCTATCAAATC-3’
BIP FP: 5’-CCTTCGATGTGCCTCTTCTCAC-3’<br>BIP RP: 5’-GGACGGGCTTCATAGTA-3’
ATF6 FP: 5’-GAACTTCGAGGATGGGTTCATAG-3’<br>ATF6 RP: 5’-GTGGTCTTGTTATGGGTGGTGGTAG-3’
SREBP1 FP: 5’-GAGCCATGGATTGCACTTTC-3’<br>SREBP1 RP: 5’-AGCATAGGGTGGGTCAAATAG-3’
L-FABP FP: 5’-GGAATGTGAGCTGGAGACAA-3’<br>L-FABP RP: 5’-AGTTCGGTCACAGACTTGATG-3’
SCD1 FP: 5’-CTCTTTCTGCTCTGCCATCTT-3’<br>SCD1 RP: 5’-CCCGACTTCACCTCCTTAAATC-3’
FASN FP: 5’-CTAGGTTTGATGCCTCCTTCTT-3’<br>FASN RP: 5’-GATGGCTTCATAGGTGACTTCC-3’
ACC FP: 5’-GCAGGTCACACGTCTCTTTAT-3’<br>ACC RP: 5’-CCAGCCTGTCATCCTCAATATC-3’
IL6 FP: 5’-GTAGTGAGGAACAAGCCAGAG-3’<br>IL6 RP: 5’-GGACTGCAGGAACTCCTTAAA-3’
TNFα FP: 5’- GAGCCAGCTCCCTCTATTTATG-3’<br>TNFα RP: 5’- AGGGCGATTACAGACACAAC-3’
TIPM2 FP: 5’-CCGAGACAAAGAGGAGAGAAAG -3’<br>TIMP2 RP: 5’-CCTCCTGATACGGGTGCCATAA -3’
Cx3Cr1 FP: 5’- GCCCTTCTGGACTCACTATTT-3’<br>Cx3Cr1 RP: 5’-CTTTGGCTTTCTTGTGGTTCTT -3’
AST FP: 5’-CGAATTTTGAGACTGGGATTAGA -3’<br>AST RP: 5’-CACTCTGGGTTGAGATGATGAA -3’
TGFβ FP: 5’- GTAAGAAGCACCAGCTTGATGT-3’<br>TGFβ RP: 5’-GTTGGAGCTGTCATCGAAGTAG -3’
p21 FP: 5’-CGCTCTACATCTTCTGCCTTAG -3’<br>p21 RP: 5’- CGGGATGAGGAGGCTTTAAATA-3’
Cytokeratin7 FP: 5’-GCTGTCCGGTGGTTAATTTC-3’<br>Cytokerytin7 RP: 5’-CAACAAGTTTTCCTCCCTCATC -3’
FGF23 FP: 5’-TAACCCATCCCTCAGCAAAC -3’<br>FGF23 RP: 5’-GAGAGGCACAAGGAAGAGAAATA -3’
BMP6 FP: 5'- GGTCTCCAGTGCTTCAGATTAC-3’<br>BMP6 RP: 5'-TGGCATCCACAAGCTCTTAC-3’
PTGS2 FP: 5'-CCAGAGCAGGCAGATGAAATA-3’<br>PTGS2 RP: 5'-CCAGTAGGCAGGAGAACATATAAC-3’
TEK FP: 5'-CAGAAGCAACAGCAACAGATAAG-3’

TEK RP: 5'-GCGGTTTGTGACTTTCCATTAG-3’
FOXL1 FP: 5'-GGCTTGGTGGTCGCTATATTT-3’<br>FOXL1 RP: 5'-ACCTCCCTCCCAACCTATTT-3’
PDLIM4 FP: 5'-GCTCCCGAGTACAGTAGTATCT-3’<br>PDLIM4 RP: 5'-TGTGCCTCCCTCATCTCTTA-3’
SIK1 FP: 5'-AGTCTCCTGTTCGCTCATAAAG-3’<br>SIK1 RP: 5'-AAGAGGTTCCACAGCAATAAGA-3’
DLGAP5 FP: 5'-CCAGACCGAGTGTTCTTTACTT-3’<br>DLGAP5 RP: 5'-CTTTGGCCTTTGACCTTGTAATC-3’
LHX2 FP: 5'-TGTTTCAGCAAGGACGGTAG-3’<br>LHX2 RP: 5'-GCCAGGCACAGAAGTTAAGA-3’
TFF2 FP: 5'-AGCTGAGCTAGACATGGGA-3’<br>TFF2 RP: 5'-GTGACACTGGAGTCGAAACA-3’
TFF1 FP: 5'-CGCCTTTGGAGCAGAGAG-3’<br>TFF1 RP: 5'-TCGAAACAGCAGCCCTTATT-3’
AVP FP: 5'-TTTGCTGCAACGACGAGA-3’<br>AVP RP: 5'-GCATTGGCGGAGGTTTATTG-3’
TEC FP: 5'-CTCCTCCGCAGTGAAGATAAAG-3’<br>TEC RP: 5'-TGGGTGCACACACCATAAA-3’
PRAMEF14 FP: 5'-CCAGGATGAATGCCTGAGATAC-3’<br>PRAMEF14 RP: 5'-GAATGATGGCACCTGGAGAA-3’
SLAMF9 FP: 5'-GCCCTTCTATGCAGATCCTAAC-3’<br>SLAMF9 RP: 5'-GCTTTGCCTCCTTCCTCAA-3’
TTC36 FP: 5'-ATTGGACCTCGGAGAGGAA-3’<br>TTC36 RP: 5'-TGGGCACGGTTGTTGTAG-3’
GPR52 FP: 5'-GCATCAGTGTGGATCGTTATCT-3’<br>GPR52 RP: 5'-CGGGCTCTTCGGTCATTTAT-3’
CAPZA3 FP: 5'-GTAATGGGCGACTACCGATTT-3’<br>CAPZA3 RP: 5'-CCAGCACTCTCCTGTTTCATAG-3’
LECT2 FP: 5'-CTGGATGCATACCCTTCTCTTC-3’<br>LECT2 RP: 5'-CCAGCGTAAGTTCTCTTGTTTATTT-3’
GSC FP: 5'-AGATGCTGCCCTACATGAAC-3’<br>GSC RP: 5'-AAACCAGACCTCCACTTTCTC-3’
TRPM1 FP: 5'-GCCTGGCGTTAGAGAAGATAC-3’<br>TRPM1 RP: 5'-GCTTGCCCTCCTCATCATATAG-3’
